# To what extent are perfusion defects seen by myocardial perfusion SPECT in patients with left bundle branch block related to myocardial infarction, ECG characteristics, and myocardial wall motion?

**DOI:** 10.1007/s12350-020-02180-7

**Published:** 2020-05-25

**Authors:** Fredrik Hedeer, Ellen Ostenfeld, Bo Hedén, Frits W. Prinzen, Håkan Arheden, Marcus Carlsson, Henrik Engblom

**Affiliations:** 1 Department of Clinical Physiology, Lund University, Skåne University Hospital, Lund, Sweden; 2 Department of Physiology, Cardiovascular Research Institute (CARIM), Maastricht University, Maastricht, The Netherlands

**Keywords:** Physiology of myocardial/coronary perfusion, physiology of LV/RV function, MRI, SPECT, gated SPECT, dyssynchrony

## Abstract

**Introduction:**

We investigated if uptake pattern on myocardial perfusion SPECT (MPS) in patients with left bundle branch block (LBBB) is related to myocardial fibrosis, myocardial wall motion, and electrocardiography (ECG) characteristics.

**Methods:**

Twenty-three patients (9 women) with LBBB, examined with MPS and cardiac magnetic resonance (CMR), were included. Tracer uptake on MPS was classified by visual interpretation as typical LBBB pattern (Defect+, n = 13) or not (Defect−, n = 10) and quantitatively. CMR images were evaluated for wall thickness and for myocardial wall motion both by visual assessment and by regional myocardial radial strain from feature tracking, and for presence and location of myocardial fibrosis. ECGs were analyzed regarding QRS duration and the presence of strict criteria for LBBB.

**Results:**

Wall thickness was slightly lower in the septum compared to the lateral wall in Defect+ patients (5.6 ± 1.1 vs 6.0 ± 1.3 mm, *P* = 0.03) but not in Defect− patients (5.6 ± 1.0 vs 5.6 ± 0.9 mm, *P* = 0.84). Defect+ patients showed a larger proportion of dyskinetic segments in the septum and hyperkinetic segments in the lateral wall compared to Defect− patients (*P* = 0.006 and *P* = 0.004, respectively). Decreased myocardial radial strain was associated with decreased tracer uptake by MPS (*R* = 0.37, *P* < 0.001). Areas of fibrosis did not match areas with uptake defect on MPS. No differences in ECG variables were seen.

**Conclusion:**

The heterogeneous regional tracer uptake in some patients with LBBB is related to underlying regional myocardial dyskinesia, wall thickening, and wall thickness rather than stress-induced ischemia, myocardial fibrosis, or specific ECG characteristics.

**Electronic supplementary material:**

The online version of this article (10.1007/s12350-020-02180-7) contains supplementary material, which is available to authorized users.

## Introduction

Left bundle branch block (LBBB) is a cardiac conduction abnormality usually seen in patients with underlying heart disease. Common causes are chronic and acute coronary artery disease, cardiomyopathy, and hypertension but LBBB even appears without any apparent underlying structural heart disease.^1^–^3^

Myocardial perfusion SPECT (MPS) is based on assessment of myocardial perfusion distribution at rest and during stress, where a fixed uptake defect at rest and stress is commonly interpreted as myocardial fibrosis or infarction.^4^ In patients with LBBB, however, tracer uptake defects are frequently seen in the septal wall.^5^–^7^ Large variability in prevalence of uptake defects on MPS in LBBB patients has been reported in different study populations.^6^ A study of 57 consecutive patients with LBBB undergoing MPS showed a prevalence of resting uptake abnormalities in the anteroseptal wall of approximately 50%.^5^ There is a risk that these patients will be falsely diagnosed with myocardial infarction. The mechanism behind the typical uptake pattern related to LBBB is still not completely understood. Several possible explanations for typical LBBB-related defects on MPS have been proposed, such as decreased septal wall thickness, myocardial wall motion abnormalities, and truly reduced blood flow due to diminished demand and/or diastolic filling time.^8^–^10^ However, it is not clear if these parameters differ in patients with LBBB and presence or absence of typical uptake pattern on MPS. Furthermore, patients with LBBB might also have myocardial fibrosis or infarction affecting the regional uptake of the tracer in the left ventricle. Thus, interpreting MPS in patients with LBBB can be a diagnostic challenge.

The aim of this study was therefore to explore the underlying pathophysiological causes of typical LBBB tracer uptake pattern on MPS in clinical patients by using cardiac magnetic resonance imaging (CMR) to assess the presence and extent of myocardial fibrosis as well as regional myocardial wall thickness and wall motion, and by assessing characteristics of the electrocardiogram (ECG) in LBBB patients with and without this typical tracer uptake pattern.

## Methods

### Study Population

Patients with LBBB who had been clinically referred to both an MPS and a CMR examination between 2002 and 2013 were retrospectively included in the study. The study protocol was approved by the regional ethics committee at Lund University (LU741/2004) and all patients had given written informed consent to participate in the study. Patient charts were studied for patient characteristics and to exclude any major adverse cardiac events occurring between the MPS and CMR examinations. In total, 23 patients fulfilled the inclusion criteria.

### Myocardial Perfusion SPECT Acquisition and Analysis

#### Image Acquisition

MPS images were acquired according to clinical routine. Patients were examined at rest and during 140 µg/kg/min adenosine stress and were injected with a weight adjusted dose of ^99m^Tc-tetrofosmin (GE Healthcare). Since gamma cameras and clinical routine changed during the years of inclusion, different protocols were used as follows:

Nine patients were examined using a Vertex dual-head gamma camera (ADAC Corporation). The ranges of ^99m^Tc-tetrofosmin were 279-668 MBq at rest and 444-1226 MBq at stress. Four of the patients underwent a 1-day rest-stress protocol, two of the patients underwent a 2-day rest-stress protocol, and three patients underwent a 2-day stress-rest protocol.

Seven patients were examined using a Ventri dual-head gamma camera (GE Healthcare). The ranges of ^99m^Tc-tetrofosmin were 639-1141 MBq at rest and 294-728 MBq at stress. Three of the patients underwent a 2-day stress-rest protocol and four of the patients underwent a 1-day stress-rest protocol.

Seven patients were examined using a solid-state cadmium zinc telluride detector gamma camera (Discovery NM 530c, GE Healthcare) and underwent a 1-day stress-rest protocol. The ranges of ^99m^Tc-tetrofosmin were 343-875 MBq at rest and 140-300 MBq at stress.

Image acquisition parameters for the Vertex gamma camera were 32 projections, steps of 5.6°, 40 s per projection, 64 × 64 matrix, and voxel size 5 × 5 × 5 mm. Iterative reconstruction using maximum likelihood-expectation maximization (MLEM) with 12 iterations was performed followed by a low-resolution Butterworth filter with a cut-off frequency set to 0.55 of Nyquist and order 5.0. Image acquisition parameters for the Ventri gamma camera were 60 projections over an orbit of 180 degrees, 20 s per projection, 64 × 64 matrix, voxel size of 6.4 × 6.4 × 6.4 mm, and a 20% symmetrical energy window over the 140 keV photon peak. Images were reconstructed with an OSEM algorithm with 12 iterations and 10 subsets, followed by post-filtration with a Butterworth filter with a cut-off frequency of 0.52 and a power of 5. Image acquisition parameters for the Discovery NM 530c gamma camera were acquisition time 480 s with a voxel size of 4 × 4 × 4 mm. Image reconstruction was performed using MLEM, 40 iterations, a Green OSL regularization alpha parameter of 0.51 and a beta of 0.3, and post-filtration with a Butterworth filter (cut-off frequency 0.37 cycles/cm and power 7).

All images were acquired in supine position approximately 45 min after the ^99m^Tc-tetrofosmin injection. ECG-gated image acquisition using 8 frames per cardiac cycle was performed at rest and stress when possible. Twenty-two patients completed both stress and rest imaging. In one patient, stress images were considered normal and rest imaging therefore was not performed. Dynamic image acquisition for quantitative perfusion assessment was not performed.

#### Image Analysis

**Figure 1:**
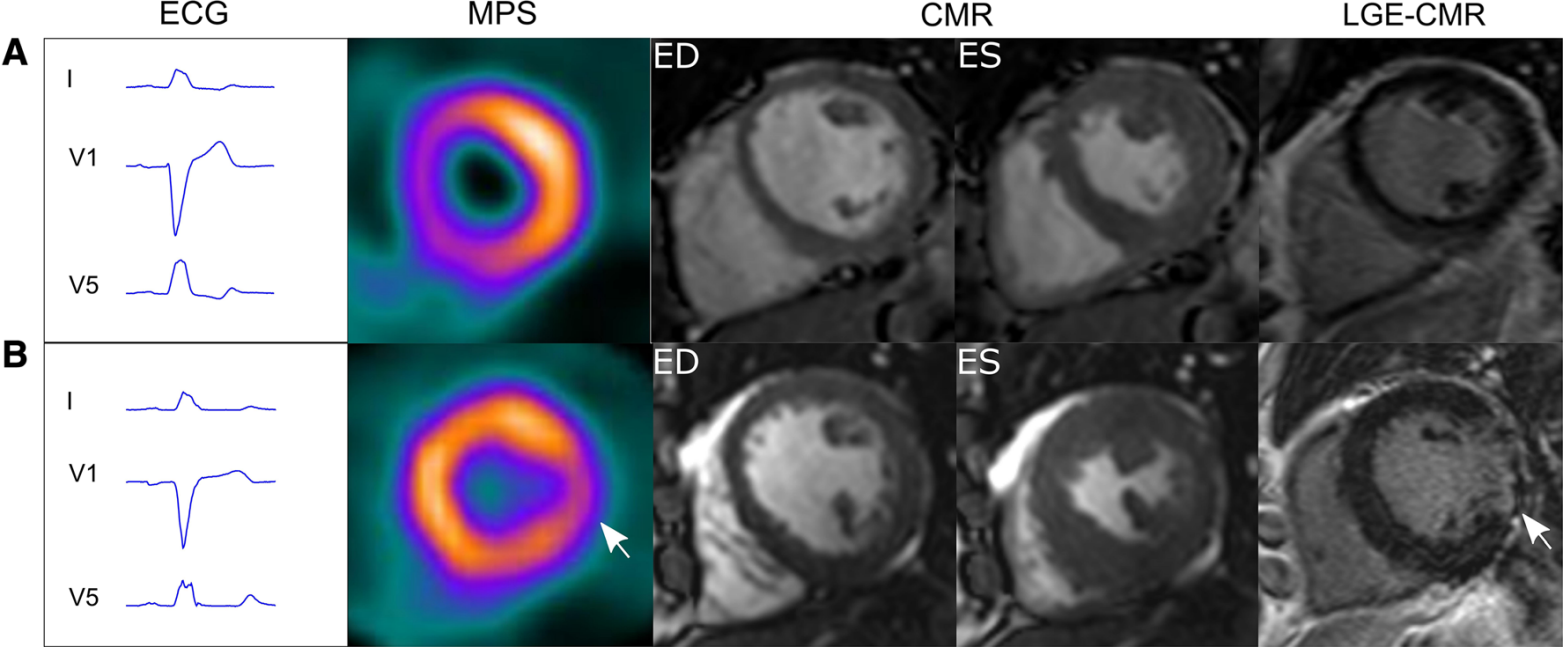
The top row **A** shows an example of a patient considered to have a typical left bundle branch block (LBBB) uptake pattern on myocardial perfusion SPECT (MPS) images (Defect+ group). One MPS short-axis slice at rest is shown. On quantitative assessment of tracer uptake on MPS, summed stress score was 19 and summed rest score was 16. The electrocardiogram (ECG) meet strict criteria for LBBB^16^ with a QRS duration of 144 ms. Cine short-axis cardiac magnetic resonance (CMR) images are shown in end-diastole (ED) and end-systole (ES). No signs of myocardial fibrosis can be found in late gadolinium enhancement (LGE)-CMR images. The bottom row **B** shows an example of a patient considered not to have a typical LBBB uptake pattern on MPS (Defect− group). The ECG meet strict criteria for LBBB with a QRS duration of 148 ms. This patient is one out of six where signs of myocardial fibrosis were found in LGE-CMR images, in this case in the endocardial parts of the basal lateral wall (arrow) constituting 3.6% of the left ventricle. One MPS short-axis slice at rest is shown. On quantitative assessment of tracer uptake on MPS, summed stress score was 3 and summed rest score was 3

Two experienced physicians analyzed the MPS images in consensus using the QPS/QGS software, version 4.0 (Cedars-Sinai, USA). The observers determined if a perfusion defect was present during stress and/or at rest and if the perfusion defect was fixed (similar during stress and at rest) or if the stress perfusion defect was greater than at rest as a sign of stress-induced ischemia. Based on the perfusion tracer uptake pattern, each patient was classified either to have an uptake pattern typical of LBBB or not. Since, to our knowledge, there is no consensus on how an MPS uptake pattern typical of LBBB should be defined, the classification was based on visual interpretation and clinical experience. A typical LBBB uptake pattern was assigned if a perfusion defect involving the septal part of the left ventricle was seen at rest and stress, not reassembling a typical LAD distribution (Figure 1a and Online Appendix). The group of patients considered to have a typical LBBB uptake pattern was called Defect+ and the group of patients with no such typical LBBB uptake pattern was called Defect−. In addition, analyses of MPS perfusion tracer uptake in 17 segments were performed both with a semi-quantitative visual analysis (summed rest score and summed stress score) and automated quantitative analysis normalized to maximum uptake as described below.

Myocardial tracer uptake was quantified by visual assessment of one experienced physician using the image analysis software Segment, version 2.2, R6338 (Medviso AB, Lund, Sweden).^11^ The left ventricle was divided into 17 segments according to the standardized model recommended by The American Heart Association (AHA).^12^ Each segment was scored using a 5-point scale ranging from 0 (normal uptake) to 4 (absent uptake). The total score of the left ventricle at stress, summed stress score (SSS), and at rest, summed rest score (SRS), as well as the summed scores of the septal (segment 2, 3, 8, and 9 in the AHA 17-segment model) and the lateral (segment 5, 6, 11, and 12 in the AHA 17-segment model) walls were calculated. In addition, automated results of perfusion at rest from QPS/QGS software, expressed as a percentage of raw counts to the maximum raw count in the image, were analyzed.

Gated MPS dyssynchrony was assessed using the automated phase analysis tool in QPS/QGS providing a phase histogram of the onset of LV myocardial contraction. Three automatically calculated parameters of LV dyssynchrony were used: phase histogram bandwidth, phase standard deviation, and entropy.^13^ For two patients, one in the Defect+ group and one in the Defect− group, no gated MPS images could be acquired and phase analysis could therefore not be performed.

### CMR Image Acquisition and Analysis

*Image acquisition:* CMR imaging was performed on a Philips Intera CV (Philips, Best, the Netherlands) with a cardiac synergy coil. All subjects were placed in supine position. Cine short-axis gradient-recalled echo images covering the left ventricle were acquired using a balanced turbo field echo (bTFE) sequence: slice thickness = 8 mm, field-of-view = 340 mm, TR = 3.14 ms, TE = 1.58 ms. In addition, three cine long-axis images (2-, 3- and 4-chamber views) were acquired using the same sequence. Approximately 15 min after intravenous administration of a commercially available extracellular gadolinium-based contrast agent (gadoteric acid, Gd-DOTA, 0.2 mmol/kg, Guerbet, Gothia Medical AB, Billdal, Sweden), an inversion-recovery (IR) sequence was used to acquire contrast-enhanced images in the corresponding planes as for the cine images. Typical IR sequence parameters were slice thickness = 8 mm, TR = 3.9 ms, TE = 1.2 ms, in-plane resolution = 1.5 x 1.5 mm, and flip angle = 15º with acquisition every heartbeat. The inversion time, typically 250-350 ms, was manually adjusted to null the signal from remote myocardium.

#### Image Analysis

The CMR images were analyzed using the software Segment.

The endocardial and epicardial borders were manually traced in each cine short-axis plane in both end-diastole and end-systole, enabling determination of left ventricular end-diastolic volume (LVEDV) and end-systolic volume (LVESV). The left ventricular ejection fraction (LVEF) was calculated as 100 × (LVEDV − LVESV)/LVEDV. Left ventricular mass (LVM) was calculated as left ventricular muscle volume multiplied by the myocardial muscle density (1.05 g/mL).

Visual assessment of myocardial wall motion was performed by one experienced physician on a segmental basis according to the AHA 17-segment model using the cine CMR short- and long-axis images. Each myocardial segment was scored according to a 5-point scale as follows: 0—hyperkinesia, 1—normokinesia, 2—hypokinesia, 3—akinesia, and 4—dyskinesia. In one patient, no cine short-axis images were acquired and therefore only long-axis images were used for the analysis. Due to image artifacts, one segment in two different patients could not be analyzed. Thus, in total 389 segments were analyzed.

Myocardial radial deformation was assessed with strain analysis using the feature tracking module in Segment.^14^ In short, mean radial strain at end-systole was quantified as follows: Endocardial and epicardial borders were delineated with exclusion of the pericardium, papillary muscles, and trabecula in end-diastole in all slices from the short-axis cine stack. Tracking points were automatically placed in a dense grid in the myocardium and propagated through the whole cardiac cycle by non-rigid image registration between adjacent time frames. Tracings were manually adjusted in end-diastole and re-propagated in case of inadequate tracking. Strain values were computed by measuring the length (*L*) between two points in end-diastole (*L*_0_) and end-systole (*L*) and determined as strain = (*L* − *L*_0_)/*L*_0_ and expressed as a percentage. Mean radial strain at end-systole was computed from the short-axis cine images and averaged on a segmental basis using the AHA 17-segment model. Since radial strain values could not be obtained for the apical segment (segment 17) in the short-axis images, 16 segments were analyzed in each patient. End-systole was defined as the time point of closure of the aortic valve as noted from the 3-chamber long-axis view. Radial strain at end-systole for the septal and lateral wall was calculated as the averaged strain values of the two basal and two mid-ventricular segments (segments 2, 3, 8, and 9 for the septal wall and segments 5, 6, 11, and 12 for the lateral wall).

Myocardial fibrosis was quantified from the short-axis late gadolinium enhancement images using full width at half maximum (FWHM) according to a previously validated method.^15^ In short, the endocardial and epicardial borders were traced manually with exclusion of the papillary muscles after which myocardial fibrosis was quantified by analyzing the signal intensity distribution within the area of interest using a FWHM approach. Manual adjustments were made when the computer algorithm delineation was inadequate. Finally, the amount of myocardial fibrosis was expressed as a percentage of the left ventricular myocardium. Subendocardial fibrosis distributed according to the coronary circulation was interpreted as being of ischemic origin, whereas non-subendocardial fibrosis not distributed according to the coronary circulation was interpreted as being of non-ischemic origin.

### ECG Acquisition and Analysis

#### ECG Acquisition

All subjects had a 12-lead ECG recorded on either a MEGACART-R (Siemens-Elema, Solna, Sweden) or an EC Sense (Cardiolex, Solna, Sweden) with a sampling rate of 500 Hz with a frequency response from 0.05 to 150 Hz.

#### ECG Analysis

The LBBB diagnosis was made by a computer algorithm made by EC Sense (Cardiolex, Solna, Sweden), version 26.3.4/1.5 (Glasgow Royal Infirmary, Glasgow, Scotland). These criteria consider several ECG characteristics: QRS slope (at multiple points), QRS (mainly R wave) morphology (in leads I, V5 and V6), Q or S morphology (in lead V1), and QRS duration and R wave amplitude (in lead V2). More specifically the criteria include QRS duration ≥ 120 ms in any two leads, large negative Q or S (in lead V1), and broad R wave with positive Q wave (in leads I, V5, V6). All ECGs were also manually analyzed to confirm the presence of LBBB using strict criteria for complete LBBB as suggested by Strauss et al.: QRS duration ≥ 140 ms for men and ≥ 130 ms for women, QS or rS in leads *V*_1_ and *V*_2_, and mid-QRS notching or slurring in ≥ 2 of leads *V*_1_, *V*_2_, *V*_5_, *V*_6_, *I*, and aVL.^16^

### Statistics

Data are presented as median (interquartile range (IQR)) or mean ± standard deviation (SD). All statistical calculations were performed using Prism 7.04 (GraphPad Software, San Diego, USA). Continuous variables were compared using *t* test, Mann–Whitney *U* test or Wilcoxon signed-rank test, and categorical variables using Fischer’s exact test. Differences between groups were tested using one-way ANOVA with post hoc testing for linear trend. A two-sided *P* value of < 0.05 was considered to indicate statistical significance.

## Results

### Patient Characteristics

Twenty-three patients were included in the study. Patient characteristics are presented in Table 1. All patients included were referred to MPS owing to suspected (n = 16) or established (n = 7) coronary artery disease. Left ventricular functional parameters were not available in two patients because complete cine CMR data were not acquired. Median time between CMR and MPS examinations was 46 (-4-70) days. According to patient charts, none of the patients had signs of major adverse cardiac events during the time between CMR and MPS examinations.

**Table 1:** Patient characteristics

	Defect+ group (n = 13)	Defect− group (n = 10)	Defect+ vs. Defect−
Age (years)	60 (55–63)	67 (57–74)	*P* = 0.14
Sex, male/female	9/4	5/5	*P* = 0.42
BMI (kg/m^2^)	25 (24–27)	28 (26–31)	*P* = 0.04
Current or former smoker	7 (54%)	7 (70%)	*P* = 0.67
Known coronary artery disease	3 (23%)	4 (40%)	*P* = 0.65
Known heart failure	6 (46%)	4 (40%)	*P* = 1.0
Atrial fibrillation	2 (15%)	0 (0%)	*P* = 0.49
Hypertension	5 (38%)	4 (40%)	*P* = 1.0
Dyslipidemia	3 (23%)	6 (60%)	*P* = 0.10
Diabetes	2 (15%)	2 (20%)	*P* = 1.0

Data are presented as median (interquartile range) or absolute number (proportion in %). *Defect+*, patients with a typical LBBB uptake pattern on MPS; *Defect*−, patients with no typical LBBB uptake pattern on MPS; *LBBB*, left bundle branch block; *MPS*, myocardial perfusion single-photon emission computed tomography; *BMI*, body mass index. No patients had pacemaker

### Myocardial Perfusion SPECT and CMR Findings

**Figure 2:**
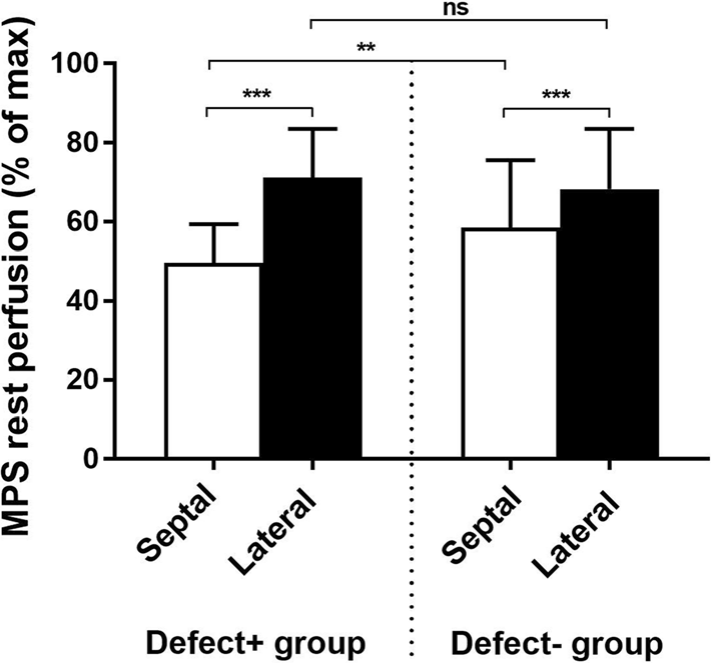
MPS rest perfusion (mean±SD), expressed as a percentage of raw counts to the maximum raw count in the image, for the septal and lateral wall in the Defect+ and Defect− group, respectively. Septal and lateral wall are the two basal and two mid-ventricular segments, segments 2, 3, 8, and 9 for the septal wall, and segments 5, 6, 11, and 12 for the lateral wall in the AHA 17-segment model. *ns*, not significant, *P < 0.05, **< 0.01 and ***P < 0.001

Polar plots of all patients are shown in the Online Appendix. Out of the 23 patients, 13 patients were considered to have a typical LBBB uptake pattern on MPS and classified to the group Defect+ (Figure 1A and Online Appendix), whereas the remaining did not have a typical LBBB uptake pattern and were classified to the group Defect− (Figure 1B and Online Appendix). Manual assessment of tracer uptake on the MPS images, SSS and SRS, differed between the Defect+ and Defect− patient groups. The Defect+ group showed significantly higher SSS and SRS in the septal wall compared to the Defect− group, whereas no differences between the groups were shown for the lateral wall (Table 2). MPS rest perfusion, expressed as a percentage of raw counts to the maximum raw count in the image, for the septal and lateral wall in the Defect+ and Defect− groups, respectively, are shown in Figure 2. There was a significantly lower MPS tracer uptake in the septal wall compared to the lateral wall in both groups (50 ± 10% vs 71 ± 12% (*P* < 0.001) and 59 ± 17% vs 68 ± 15% (*P* < 0.001) for the Defect+ and Defect− group, respectively). The tracer uptake in the septal wall was significantly lower in the Defect+ group compared to the Defect− group (*P* = 0.002), whereas no difference in tracer uptake between the groups was shown in the lateral wall (*P* = 0.32).

**Table 2:** Myocardial perfusion SPECT results

	Defect+ group (n = 13)	Defect− group (n = 10)	Defect+ vs. Defect−
SSS on MPS			
Entire LV	17 (9–23)	8 (5–16)	*P* = 0.02
Septal wall	7 (4–9)	4 (1–5)	*P* = 0.003
Lateral wall	0 (0–1)	0 (0–1)	*P* = 1.0
SRS on MPS			
Entire LV	17 (13–20)	10 (5–16)	*P* = 0.02
Septal wall	7 (6–8)	5 (3–6)	*P* = 0.003
Lateral wall	0 (0–1)	0 (0–2)	*P* = 0.91

Data are presented as median (interquartile range). *Defect*+, patients with a typical LBBB uptake pattern on MPS; *Defect*−, patients with no typical LBBB uptake pattern on MPS; *LBBB*, left bundle branch block; *MPS*, myocardial perfusion single-photon emission computed tomography; *SSS*, summed stress score; *SRS*, summed rest score (0—normal uptake, 4—absent uptake). *LV*, left ventricle. In the patient group with no typical LBBB uptake pattern on MPS, one patient did not complete MPS at rest since the stress examination was considered normal

One patient in the Defect− group was diagnosed to have stress-induced ischemia based on the MPS images, whereas the other 22 patients did not have ischemia.

#### Myocardial Fibrosis

Six of the 23 patients showed signs of myocardial fibrosis on LGE-CMR, four patients in the Defect+ group and two patients in the Defect− group.

In the Defect+ group, three of the four patients had fibrosis interpreted as of non-ischemic origin, whereas one patient had fibrosis interpreted as of ischemic origin. Two of these patients had parts of their fibrosis in the septal wall. Fibrosis quantification showed fibrosis volumes ranging from 0.7 to 3% of the left ventricle in these four patients. In none of the cases, the distribution or the extent of myocardial fibrosis on CMR corresponded to the distribution or extent of uptake defects on MPS.

In the Defect− group, both patients had fibrosis in the lateral wall constituting 3.6% and 9.1% of the left ventricle, respectively, and the fibrosis was interpreted as of ischemic origin (Figure 1B).

When comparing patients with and without fibrosis on LGE-CMR, there were no significant differences in SSS or SRS, for the entire left ventricle (*P* = 0.80 for SSS and *P* = 0.41 for SRS), for the septal wall (*P* = 0.52 for SSS and *P* = 0.48 for SRS), or for the lateral wall (*P* = 0.07 for SSS and *P* = 0.33 for SRS).

#### Left Ventricular Mass, Wall Thickness, and WALL Motion

Global left ventricular function was similarly depressed in both patient groups, with a mean EF of 35%. No differences in left ventricular mass, volumes, and ejection fraction by CMR were seen between the Defect+ and Defect− groups (Table 3).

**Table 3:** Cardiac magnetic resonance and electrocardiography results

	Defect+ group (n = 13)	Defect− group (n = 10)	Defect+ vs. Defect−
CMR			
LGE (n)	4 (31%)	2 (20%)	*P* = 0.66
LVM (g)	104 (87–155)	110 (88–139)	*P* = 0.90
LVEDV (mL)	222 (202–304)	272 (171–281)	*P* = 0.75
LVESV (mL)	148 (112–222)	165 (77–219)	*P* = 0.74
LVEF (%)	34 (24–44)	35 (26–57)	*P* = 0.33
Mean wall thickness in end-diastole (mm)			
Septal wall	5.6 ± 1.1	5.6 ± 1.0	*P* = 0.88
Lateral wall	6.0 ± 1.3	5.6 ± 0.9	*P* = 0.54
Radial strain entire LV (%)	24 ± 12	33 ± 18	*P* = 0.20
Radial strain septal wall (%)	6 ± 17	17 ± 25	*P* = 0.02
Radial strain lateral wall (%)	38 ± 17	42 ± 24	*P* = 0.39
Difference in radial strain between lateral and septal wall (%)	32 ± 18	25 ± 29	*P* = 0.18
ECG			
Strict LBBB criteria met (n)	12 (92%)	9 (90%)	*P* = 1.0
QRS duration (ms)	152 (142–162)	156 (143–162)	*P* = 0.87
Maximum HR at adenosine stress (bpm)	95 (87–105)	97 (82–109)	*P* = 0.93

Data are presented as median (interquartile range), mean ± SD, or absolute number (proportion in %)

*Defect*+, patients with a typical LBBB uptake pattern on MPS; *Defect*−, patients with no typical LBBB uptake pattern on MPS; *LBBB*, left bundle branch block; *MPS*, myocardial perfusion single-photon emission computed tomography; *CMR*, cardiac magnetic resonance; *LGE*, fibrosis assessed with late gadolinium enhancement; *LV*, left ventricle; *LVM*, left ventricular mass; *LVEDV*, left ventricular end-diastolic volume; *LVESV*, left ventricular end-systolic volume; *LVEF*, left ventricular ejection fraction; *ECG*, electrocardiography; *HR*, heart rate. *bpm*, beats per minute. In two patients, one in each group, analysis of left ventricular mass, volumes, and wall thickness could not be performed. In one patient in the group with no typical LBBB uptake pattern on MPS, analysis of radial strain could not be performed. In two patients in the group with no typical LBBB uptake pattern on MPS, no data on maximum heart rate at adenosine stress could be obtained retrospectively

There was a small but systematic difference in wall thickness between the septal and lateral wall. Patients in the Defect+ group had a significantly lower mean end-diastolic thickness in the septal wall compared to the lateral wall (difference 0.4 ± 0.6 mm, *P* = 0.03), whereas patients in the Defect− group had not (difference 0.0 ± 0.6 mm, *P* = 0.84).

**Figure 3:**
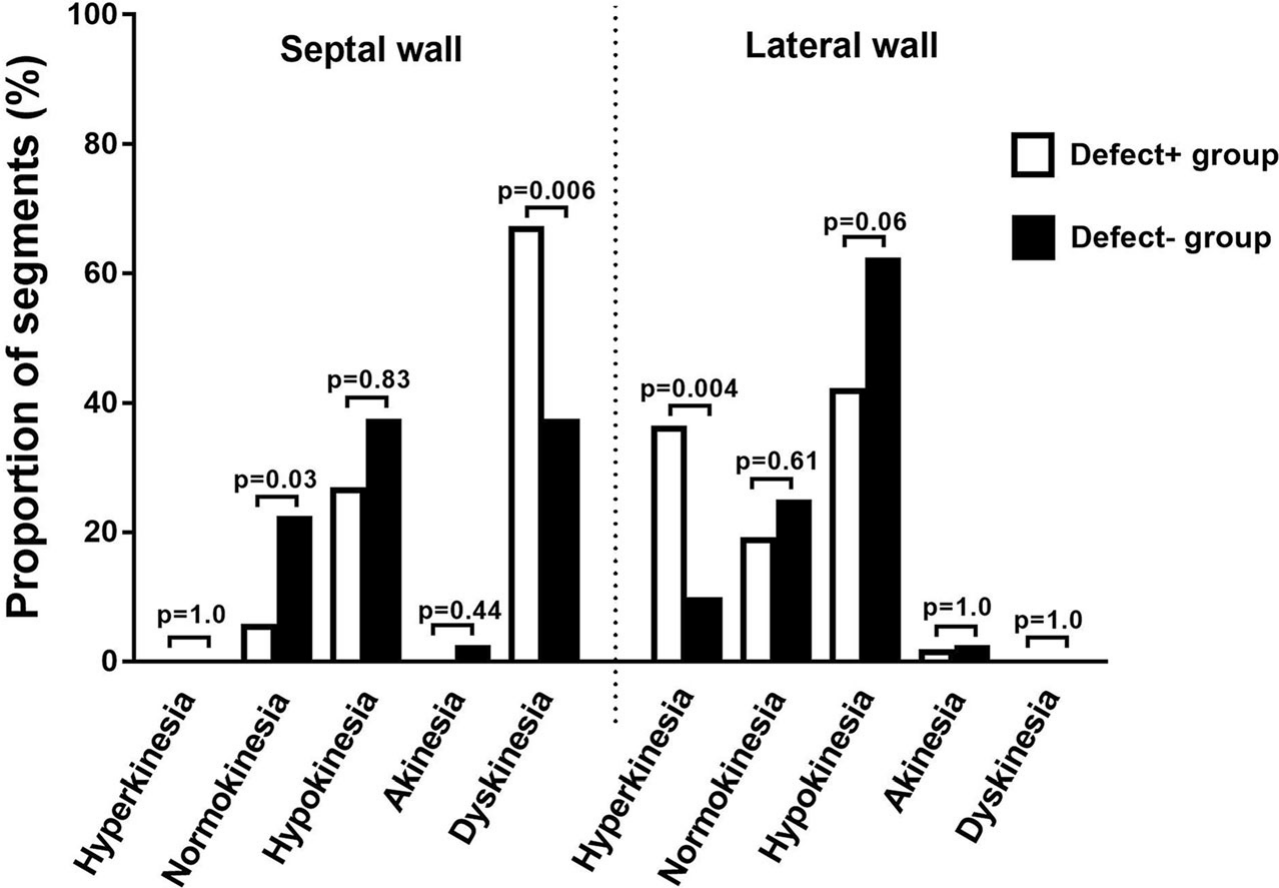
Visual assessment of regional left ventricular wall motion on CMR according to the AHA 17-segment model, where wall motion in each segment was graded to one of the following: hyperkinesia, normokinesia, hypokinesia, akinesia, or dyskinesia. Patients with a typical left bundle branch block (LBBB) uptake pattern on myocardial perfusion SPECT (MPS) (white bars), Defect+ group, and patients with no typical LBBB uptake pattern on MPS (black bars), Defect− group

Results of visual assessment of regional left ventricular wall motion are shown in Figure 3. The Defect+ group showed a larger proportion of dyskinetic segments in the septal wall (*P* = 0.006) and a larger proportion of hyperkinetic segments in the lateral wall (*P* = 0.004) compared to the Defect− group.

Results of gated MPS phase analysis are shown in Table 4. Values of entropy, phase histogram bandwidth, and phase standard deviation were slightly higher for the Defect+ group compared to the Defect− group, but the differences were not statistically significant (*P* = 0.17, *P* = 0.26 and *P* = 0.20, respectively).

**Table 4:** Gated MPS phase analysis

	Defect+	Defect−	Defect+ vs. defect−
Entropy (%)	51 (48–62)	45 (42–62)	*P* = 0.17
Phase histogram bandwidth (°)	57 (48–84)	42 (32–105)	*P* = 0.26
Phase standard deviation (°)	15 (12–22)	10 (9–27)	*P* = 0.20

Data presented as median (interquartile range). *MPS*, myocardial perfusion single-photon emission computed tomography; *Defect*+ patients with a typical LBBB uptake pattern on MPS; *Defect*−, patients with no typical LBBB uptake pattern on MPS

**Figure 4:**
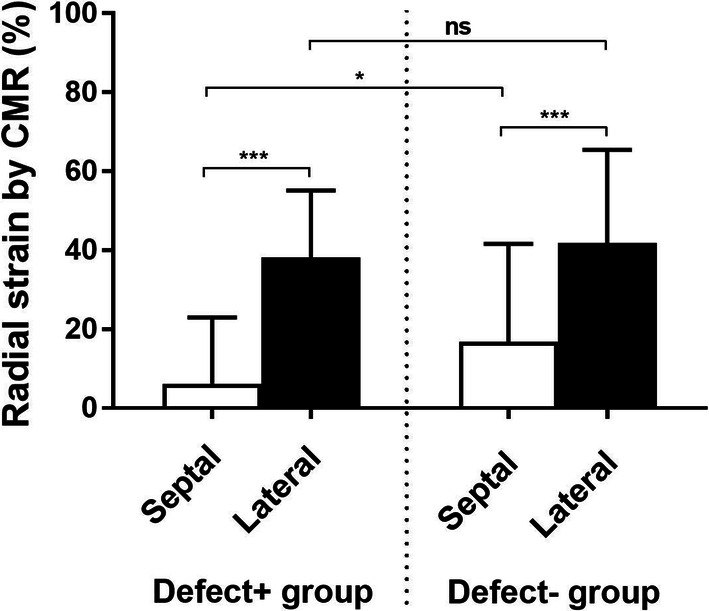
Radial strain (mean±SD) at end-systole in cardiac magnetic resonance short-axis images, for the septal and lateral wall in the Defect+ and Defect− group, respectively. Septal and lateral wall are the two basal and two mid-ventricular segments, segments 2, 3, 8, and 9 for the septal wall, and segments 5, 6, 11, and 12 for the lateral wall in the AHA 17-segment model. *ns*, not significant, *P < 0.05, **P < 0.01, and ***P < 0.001

Results of left ventricular radial strain values are shown in Table 3 and Figure 4. Radial strain values at end-systole were lower in the septal wall compared to the lateral wall both in the Defect+ group and in the Defect− group (6 ± 17% vs. 38 ± 17% (*P* < 0.001) and 17 ± 25% vs. 42 ± 24% (*P* < 0.001) for the Defect+ and Defect− group, respectively). Septal wall radial strain was lower in the Defect+ group compared to the Defect− group (*P* = 0.02), whereas lateral wall radial strain did not differ between the groups (*P* = 0.39).

**Figure 5:**
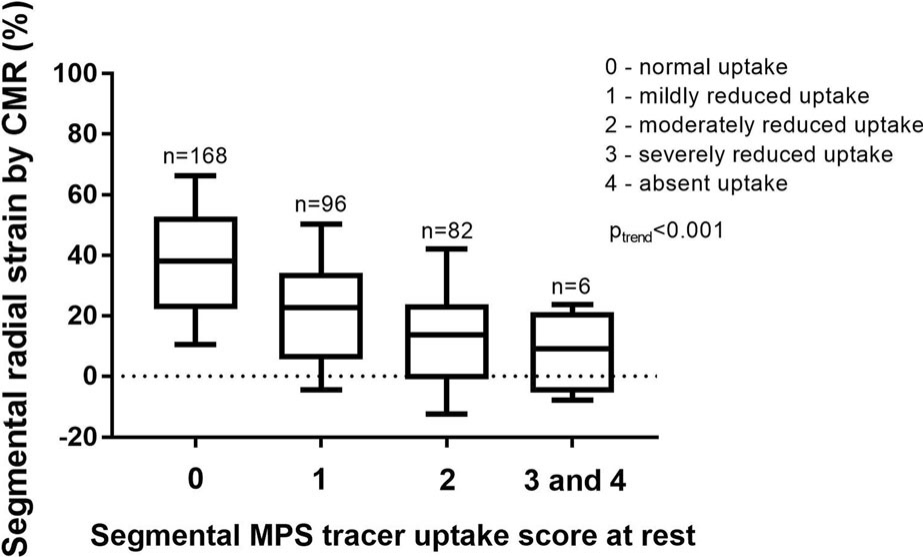
Radial strain on cardiac magnetic resonance (CMR) images in each segment according to the AHA 17-segment model in all patients, correlated to visual assessment of uptake score on myocardial perfusion SPECT (MPS) images at rest in each segment in all patients. Boxes extend from 25th to 75th percentiles and whiskers extend from 10th to 90th percentiles. In total, 352 segments could be analyzed. One patient did not complete rest MPS examination since the stress MPS examination was considered normal. For this patient, the MPS tracer uptake scores at stress are used. n = number of segments in each MPS tracer uptake score group

**Figure 6:**
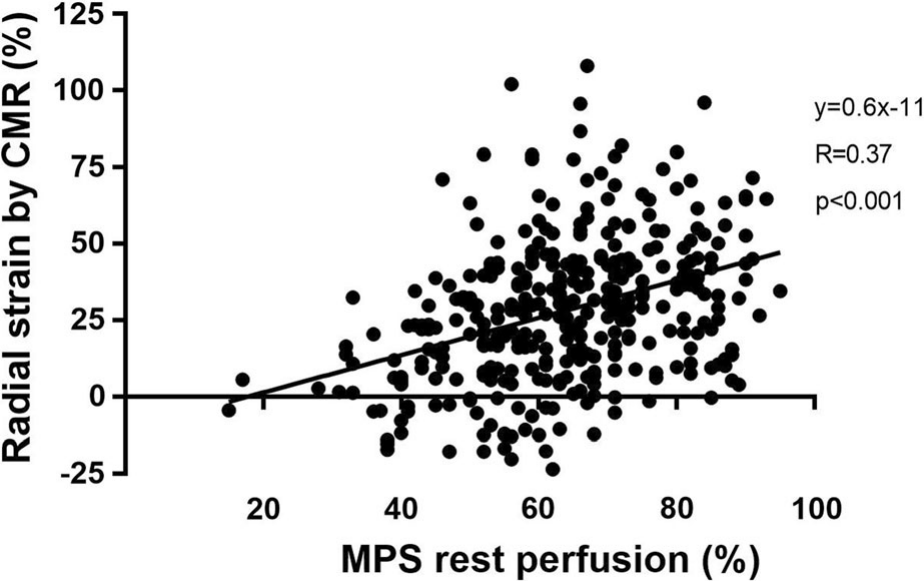
Relationship between MPS tracer uptake, expressed as a percentage of raw counts to the maximum raw count in the image, and left ventricular radial strain by CMR in all patients on a myocardial segmental level. In total, 352 segments were analyzed. One patient did not complete rest MPS examination since the stress MPS examination was considered normal. For this patient, the MPS tracer uptake scores at stress are used

Figure 5 shows the relationship between MPS tracer uptake score at rest and left ventricular radial strain by CMR in all patients on a myocardial segmental level according to the AHA 17-segment model. Radial strain values were significantly higher in segments with normal tracer uptake compared to segments with reduced tracer uptake (*P* < 0.001). There was a trend of gradually lower strain values in segments with gradually lower tracer uptake (higher uptake score) (*P* < 0.001 for trend). The relationship between MPS tracer uptake and left ventricular radial strain by CMR is shown in Figure 6.

### ECG Findings

Twenty-one of 23 patients diagnosed with LBBB by the conventional criteria also met strict LBBB criteria according to Strauss et al^16^ (Table 3). One male patient in the Defect+ group had a QRS duration of 136 ms (i.e., < 140 ms) but fulfilled the other two criteria. This patient had an LVM of 102 g which was the lowest LVM in the Defect+ group. One female patient in the Defect− group had a QRS duration of 128 ms (i.e., < 130 ms) but fulfilled the other two criteria. This patient had an LVM of 79 g which was the lowest LVM in the Defect− group. There were no differences in QRS duration or maximum heart rate at adenosine stress between the Defect+ and Defect− groups (Table 3).

A summary of MPS, CMR and ECG results for each individual patient is presented in Table 5.

**Table 5:** Summary of MPS, CMR, and ECG results for each patient

Patient (gender)	MPS			CMR			ECG	
	Typical LBBB uptake pattern	SSS	SRS	Fibrosis (LGE-CMR)	Strain_sept_ (%)	Strain_lat_ (%)	Strict LBBB criteria met	QRS duration (ms)
1 (F)	+	18	24	2.2%, ant, ischemic	12	34	Yes	152
2 (M)	+	23	28	No	21	47	Yes	160
3 (M)	+	21	20	No	− 6	10	Yes	164
4 (M)	+	20	18	No	− 3	40	Yes	164
5 (M)	+	17	15	No	8	43	Yes	144
6 (M)	+	17	19	3%, ant, non-ischemic	− 1	34	Yes	158
7 (F)	+	13	14	No	− 15	41	Yes	172
8 (M)	+	15	18	2%, sept, non-ischemic	− 11	17	Yes	156
9 (F)	+	16	17	No	9	42	Yes	138
10 (M)	+	9	8	0.7%, sept, non-ischemic	6	29	No	136
11 (F)	+	10	10	No	7	58	Yes	140
12 (M)	+	12	15	No	22	36	Yes	148
13 (M)	+	19	16	No	30	65	Yes	144
14 (M)	−	21	20	9.1%, lat, ischemic	18	11	Yes	180
15 (F)	−	8	10	No	-1	43	Yes	174
16 (F)	−	2	*	No	55	62	No	128
17 (M)	−	15	15	No	2	19	Yes	158
18 (M)	−	3	3	3.6%, lat, ischemic	32	41	Yes	146
19 (M)	−	5	3	No	− 3	53	Yes	156
20 (F)	−	12	10	No	n/a	n/a	Yes	156
21 (F)	−	8	9	No	30	82	Yes	146
22 (M)	−	5	6	No	27	40	Yes	158
23 (F)	−	18	16	No	− 10	26	Yes	132

Data are presented as absolute values

*MPS*, myocardial perfusion single-photon emission computed tomography; *CMR*, cardiac magnetic resonance;* ECG*, electrocardiogram; *LBBB*, left bundle branch block; *SSS*, summed stress score; *SRS*, summed rest score; *LGE*, late gadolinium enhancement; *F*, female; *M*, male; *Sept*, ventricular septal wall; *Lat*, left ventricular lateral wall; *Ant*, left ventricular anterior wall. *no rest imaging was performed since stress imaging was considered normal

## Discussion

The findings in the present study suggest that in patients with LBBB, the presence of typical LBBB uptake pattern on MPS is related to regional myocardial dyskinesia, wall thickening, and wall thickness rather than stress-induced ischemia, myocardial fibrosis, or characteristics on ECG.

Thirteen out of 23 patients with LBBB showed a typical LBBB uptake pattern on MPS. These results are consistent with our clinical experience that tracer uptake on MPS images in patients with LBBB vary considerably. The results for patients with typical LBBB uptake pattern on MPS are consistent with the findings by Mahrholdt et al.^9^ However, in the study by Mahrholdt et al all patients were reported to have septum-related defects on MPS, whereas our study also included patients with LBBB with no typical LBBB uptake pattern on MPS. The prevalence of typical LBBB uptake pattern on MPS was similar to the results found by Alexanderson et al.^5^

As expected, tracer uptake was quantified significantly lower in the septal wall compared to the lateral wall in the Defect+ patients. However, a similar pattern was also seen in the Defect− patients, although they were judged to have a tracer uptake pattern not typical of LBBB. This is reflecting a continuum from normal tracer distribution and wall motion to a distinct septal-lateral gradient of both tracer uptake and regional wall motion among LBBB patients. The polar plots of the patients at the Online Appendix show this range of decreased perfusion tracer uptake.

In the present study, visual assessment of regional left ventricular wall motion on CMR showed that patients with typical LBBB uptake pattern on MPS had a larger proportion of dyskinetic segments in the septal wall and a larger proportion of hyperkinetic segments in the lateral wall compared to patients with no typical LBBB uptake pattern on MPS. Additionally, analysis of myocardial deformation in the left ventricle by assessment of myocardial radial strain on CMR images showed a difference in strain values between myocardial segments with normal tracer uptake and segments with reduced tracer uptake on MPS. These results are in agreement with those of Vernooy et al^10^ who studied a canine model before and after induction of LBBB, where myocardial wall shortening and myocardial mechanical work decreased in the septal wall and increased in the lateral wall after onset of LBBB. The results are also in concordance with Mahrholdt et al^9^ who presented that the dyskinetic myocardial wall segments showed low tracer uptake on MPS to a greater extent than myocardial wall segments with normal motion. Furthermore, we show that this mechanism is valid even in our study population consisting of LBBB patients both with and without typical LBBB uptake pattern on MPS. The strain pattern presented in our study, with lower radial strain in the septal wall compared to the lateral wall of the left ventricle, is consistent with findings in previous studies of strain pattern in dyssynchrony.^17^,^18^ There was a larger proportion of hyperkinetic segments, found by visual analysis, in the lateral wall in the Defect+ group compared to the Defect− group. Unexpectedly, there was no difference in mean radial strain in the lateral wall between the Defect+ and Defect− group. A possible explanation could be differences in statistical analysis where the visual analysis was qualitative, based on categories, whereas strain analysis resulted in continuous data. Another possible explanation could be that visual analysis takes into account the whole cardiac cycle, whereas radial strain analysis was predefined to end-systolic strain.

Slightly higher values for gated MPS phase analysis parameters (phase histogram bandwidth, phase standard deviation, and entropy) were shown in the Defect+ group compared to the Defect− group but the differences were not statistically significant. Higher values for these parameters have been shown in patients with LBBB compared to patients with no conduction abnormalities.^19^ Interestingly, one study investigating MPS phase analysis in patients with and without myocardial scar reported differences between the groups of the same magnitude as the ones in the current study.^20^ The number of patients in the current study is, however, too small to be able to compare differences of this magnitude for MPS phase analysis parameters, given the inter-individual variability.

Besides the dyskinetic wall motion, a true change in myocardial perfusion is another pathophysiologic mechanism proposed to explain the typical uptake pattern on MPS in patients with LBBB.^6^ Vernooy et al^10^ reported that myocardial blood flow, measured by microspheres in an experimental model, decreased in the septal wall and increased in the lateral wall after onset of LBBB. Patients with LBBB, however, were shown to have an increased absolute myocardial blood flow in the lateral wall but not a decrease in the septal wall at stress, when assessed by ^15^O-H_2_O positron emission tomography.^21^ We found increased regional function in the lateral wall in patients with Defect+ in line with increased myocardial blood flow in the lateral wall on PET. Assessment of absolute myocardial perfusion was, however, not available in our retrospective data.

Experimental work has shown the temporal evolution of LBBB-induced work and glucose metabolism.^22^ Early differences with higher myocardial work and glucose metabolism in the lateral wall compared to the septal wall disappeared after 8 weeks of pacing. Thus, another possible mechanism to why some LBBB patients have septal to lateral wall inhomogeneity on MPS whereas others do not may be differences between patients in adaptation. Data on the timing of LBBB onset were, however, not available for the present study population.

In myocardial perfusion SPECT, the static perfusion images reflect a cumulative amount of gamma radiation counts from repeated cardiac cycles in both diastole and systole. As the spatial resolution of SPECT is limited, relatively more counts will be detected in the systolic frames during myocardial wall thickening and relatively less counts will be detected in diastole when the myocardial wall is thinner. Additionally, a dyskinetic segment will change position within the SPECT matrix during the cardiac cycle which implies that the cumulative amount of counts for each voxel will be lower and the detected counts being “blurred.” These mechanisms could be parts of the explanation of why LBBB patients with dyskinetic septal myocardial segments and hyperkinetic lateral myocardial segments in our study showed lower MPS tracer uptake in the septal parts compared to the lateral parts of the left ventricle.

The Defect+ group had a slightly thinner septal wall compared to the lateral wall, but this difference was not found in the Defect− group. The difference in wall thickness between the septal and lateral wall in the Defect+ group was, however, small (mean 0.4 mm). Previous studies^9^,^23^ have shown that thin myocardial wall segments in patients with LBBB are associated with tracer uptake defects in the SPECT images. Hassan et al^8^ showed in phantom studies that due to partial volume effect and limited spatial resolution, SPECT underestimates the maximal voxel activity in small structures. For example, a decrease in phantom tube size of 3.5 mm (corresponding to a decrease in myocardial wall thickness between end-systole and end-diastole) was related to a decrease in maximal voxel activity of 38%. A difference in wall thickness of 0.4 mm, found in the present study, would translate to a difference in counts of approximately 10%-15% between the lateral and septal wall. Thus, limited spatial resolution in combination with thin myocardium is not likely to be a major explanation to the typical uptake pattern on MPS in some LBBB patients found in this study.

We found six patients with fibrosis on LGE-CMR. In none of the cases in the Defect+ group, the distribution or the extent of myocardial fibrosis on CMR corresponded to the distribution or extent of uptake defects on MPS. Thus, the typical LBBB uptake pattern on MPS could not be explained by myocardial fibrosis in the present study. However, cases with infarction in the lateral wall have been shown to have improved septal function^24^ and hence could possibly counteract the typical uptake pattern in LBBB. Since we found only two patients with myocardial fibrosis in the lateral wall in the Defect− group, this was not the mechanism in the majority of patients.

An additional explanation could be that differences in QRS duration may explain why some LBBB patients but not all have typical uptake pattern on MPS. The prolonged QRS duration is a measure of conduction abnormality and can be related to ventricular dyssynchrony.^25^ Such a relationship could not be found in our study. In contrast, a study by Inanir et al^26^ found a correlation between minimum QRS duration and severity of MPS perfusion abnormalities in patients with LBBB. They also chose to evaluate the maximum and mean QRS durations and they found no correlation between mean QRS duration, consistent with our study, or maximum QRS duration and uptake defects on MPS images. Inanir et al excluded all patients with prior myocardial infarction and known coronary artery disease, and they regarded the perfusion as normal if SSS on the MPS images was equal to or less than 3. Thus, the studies differ in inclusion criteria and definition of perfusion defects.

Furthermore, LBBB patients with high heart rate have been shown to have more uptake defects on MPS than patients with lower heart rate.^7^,^27^ In our study, there were no differences in maximum heart rate at adenosine stress between patients in the Defect+ and Defect− groups. Thus, differences in maximum heart rate could not explain the findings in this study.

Two out of 23 patients did not fulfill the strict Strauss ECG criteria for LBBB, one in the Defect+ group and one in the Defect− group, both due to a QRS duration just below the lower limit. These two patients had the lowest LVM in the Defect+ and the Defect− groups, respectively, which could explain the slightly shorter QRS duration.^28^

In a clinical context, it is important for an MPS interpreting physician to acknowledge that patients with LBBB might or might not have a septum-related tracer uptake defect caused by the LBBB. If an uptake defect is present, it is likely not caused by myocardial fibrosis or ischemia, but rather related to myocardial dyskinesia, wall thickening, and wall thickness. However, if for clinical reasons myocardial fibrosis must be excluded, further examination with LGE-CMR is recommended.

## Limitations

The main limitation of this study is the low number of patients owing to the retrospective design including only patients with both CMR and MPS examinations from clinical referrals. Some patients had several days between the MPS and CMR examinations which theoretically could affect the comparison of MPS tracer uptake and myocardial function assessed on CMR. However, no clinical events were noted in the clinical records. Furthermore, six patients had their CMR examinations prior to the MPS examination. In those cases, an eventual myocardial infarction occurring after the CMR and before the MPS examination may result in myocardial fibrosis which can affect the MPS images but would not be present in the CMR images. However, the majority of the patients underwent the CMR examination the same day or after the MPS examination, why this issue was non-existent. Of note, according to patient charts none of the patients had signs of major adverse cardiac events during the time between the MPS and CMR examinations. A limitation is that the classification of patients to have an MPS tracer uptake pattern typical of LBBB or not was based on visual interpretation and clinical experience. To the best of our knowledge, there is no consensus on how an MPS uptake pattern typical of LBBB should be defined and the descriptions of LBBB uptake pattern in the literature are often scant and not precise. Therefore, we based our definition of an MPS uptake pattern typical of LBBB on visual interpretation and clinical experience. Furthermore, results of MPS perfusion quantification, MPS segmental rest score as well as a continuous variable of automated MPS segmental rest perfusion %, are also shown for the whole study population, without the Defect+ and Defect− classification. Another limitation is that different SPECT cameras and different MPS protocols were used, due to change in clinical routines and clinical SPECT cameras during the years of inclusion. Finally, the patients were not examined with an independent method for assessing the presence or absence of myocardial ischemia.

## Conclusion

The typical uptake pattern seen on MPS in some patients with LBBB is likely related to underlying regional myocardial dyskinesia, wall thickening, and wall thickness rather than stress-induced ischemia, myocardial fibrosis, or specific ECG characteristics.

## New Knowledge Gained

In a patient cohort with LBBB, containing both patients with and without typical LBBB uptake pattern on MPS, we show a relation between decreased uptake on MPS and regional myocardial wall motion, thickening, and thickness assessed on CMR images, both by visual analysis and regional myocardial radial strain measurements.

## Electronic supplementary material

Below is the link to the electronic supplementary material.

Electronic supplementary material 1 (PDF 863 kb)

Electronic supplementary material 2 (DOCX 317 kb)

## Abbreviations

LBBB: Left bundle branch block
MPS: Myocardial perfusion single-photon emission computed tomography
CMR: Cardiac magnetic resonance
LGE: Late gadolinium enhancement
ECG: Electrocardiogram
