# Supplementary material for: To what extent are perfusion defects seen by myocardial perfusion SPECT in patients with left bundle branch block related to myocardial infarction, ECG characteristics, and myocardial wall motion?
Source: J Nucl Cardiol. 2020 May 25;28(6):2910–22. doi: 10.1007/s12350-020-02180-7 (PMC8709823; doi:10.1007/s12350-020-02180-7)
Supplement: Supplementary file 1 — Electronic supplementary material 1 (PDF 863 kb) [file 12350_2020_2180_MOESM1_ESM.pdf]

Journal of Nuclear Cardiology | Official Journal of the American Society of Nuclear Cardiology

To what extent are perfusion defects seen by myocardial perfusion SPECT in patients with left bundle branch block related to myocardial infarction, ECG characteristics and myocardial wall motion?

Fredrik Hedeer, MD[1], Ellen Ostenfeld, MD, PhD[1], Bo Hedén, MD, PhD[1], Frits W. Prinzen, PhD[2], Håkan Arheden, MD, PhD[1], Marcus Carlsson, MD, PhD[1], Henrik Engblom, MD, PhD[1]

[1]Lund University, Skåne University Hospital, Department of Clinical Physiology, Lund, Sweden
[2]Department of Physiology, Cardiovascular Research Institute (CARIM), Maastricht University, Maastricht, the Netherlands

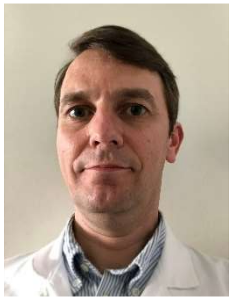

LUND UNIVERSITY

For Peer Review

Copyright American Society of Nuclear Cardiology

Journal of Nuclear Cardiology | Official Journal of the American Society of Nuclear Cardiology

BACKGROUND

The aim of this study was to explore the underlying pathophysiological causes of typical LBBB tracer uptake pattern on MPS in clinical patients by using cardiac magnetic resonance imaging (CMR) to assess presence and extent of myocardial fibrosis as well as regional myocardial wall thickness and wall motion, and by assessing characteristics of the electrocardiogram (ECG) in LBBB patients with and without this typical tracer uptake pattern.

For Peer Review

Copyright American Society of Nuclear Cardiology

Journal of Nuclear Cardiology | Official Journal of the American Society of Nuclear Cardiology

METHODS

A. Study type: Retrospective.

B. Study subjects: 23 patients (9 women) who had undergone myocardial perfusion SPECT (MPS) and cardiac magnetic resonance (CMR) on clinical indication were retrospectively included.

C. Study endpoints: Explorative study.

D. Study variables: LBBB-related uptake pattern or not (+/-) on MPS. Quantification of tracer uptake on MPS. Quantification of wall motion by visual and strain analysis on CMR. ECG characteristics.

For Peer Review

Copyright American Society of Nuclear Cardiology

Journal of Nuclear Cardiology | Official Journal of the American Society of Nuclear Cardiology

RESULTS

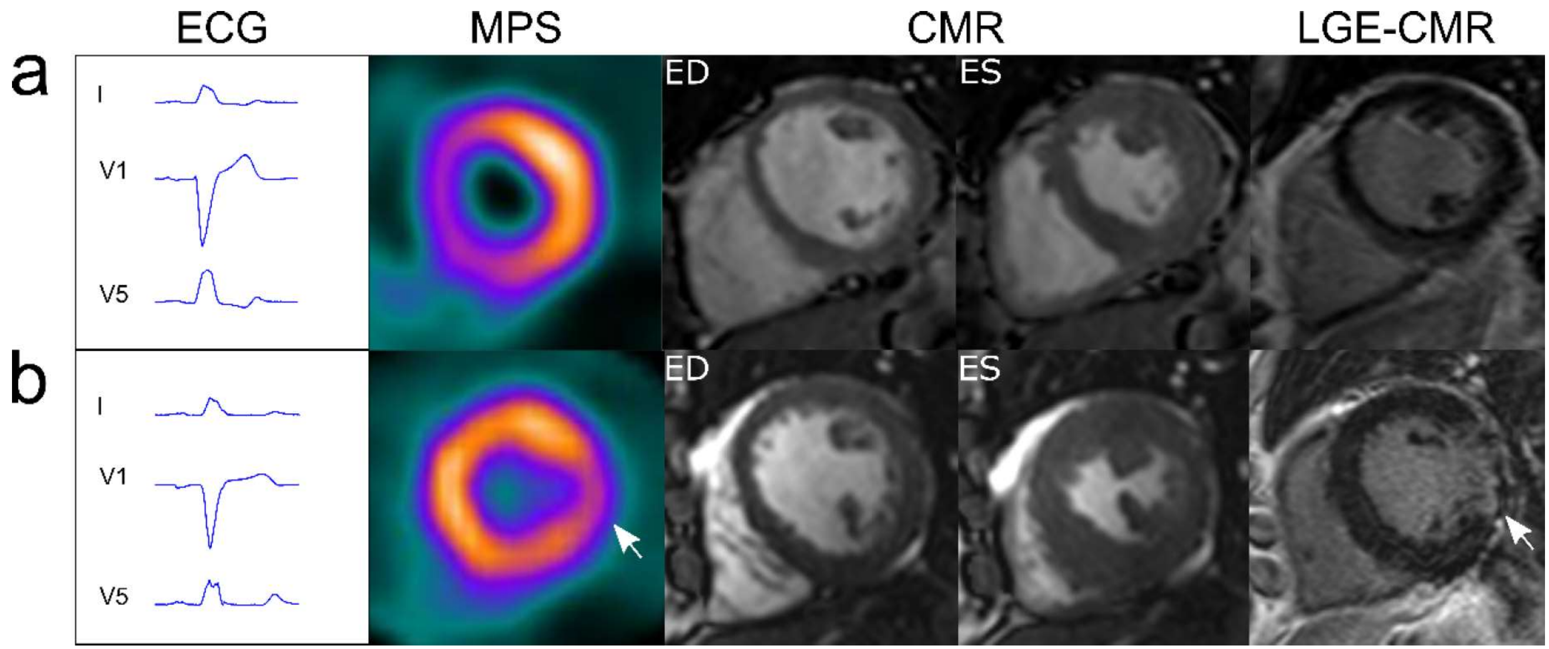

The top row (*a*) shows an example of a patient considered to have a typical left bundle branch block (LBBB) uptake pattern on myocardial perfusion SPECT (MPS) images (Defect+ group). One MPS short axis slice at rest is shown. On quantitative assessment of tracer uptake on MPS, summed stress score was 19 and summed rest score was 16. The electrocardiogram (ECG) meet strict criteria for LBBB according to Strauss et al. with a QRS duration of 144 ms. Cine short axis cardiac magnetic resonance (CMR) images are shown in end-diastole (ED) and end-systole (ES). No signs of myocardial fibrosis can be found in late gadolinium enhancement (LGE)-CMR images. The bottom row (*b*) shows an example of a patient considered not to have a typical LBBB uptake pattern on MPS (Defect- group). The ECG meet strict criteria for LBBB with a QRS duration of 148 ms. One MPS short axis slice at rest is shown. This patient is one out of six where signs of myocardial fibrosis were found in LGE-CMR images, in this case in the endocardial parts of the basal lateral wall (arrows) constituting 3.6 % of the left ventricle. On quantitative assessment of tracer uptake on MPS, summed stress score was 3 and summed rest score was 3.

Copyright American Society of Nuclear Cardiology

Journal of Nuclear Cardiology | Official Journal of the American Society of Nuclear Cardiology

RESULTS

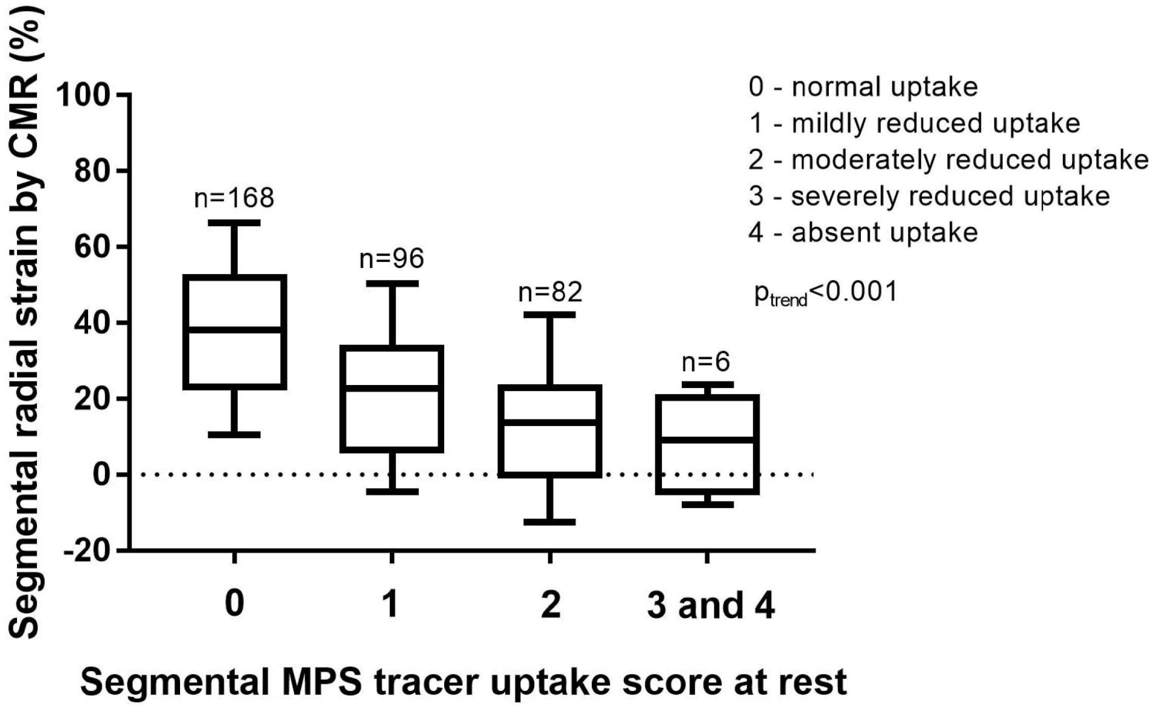

Radial strain on cardiac magnetic resonance (CMR) images in each segment according to AHA 17-segment model in all patients, correlated to visual assessment of uptake score on myocardial perfusion SPECT (MPS) images at rest in each segment in all patients. Boxes extends from 25th to 75th percentiles and whiskers extends from 10th to 90th percentiles. In total, 352 segments could be analyzed. One patient did not complete rest MPS examination since the stress MPS examination was considered normal. For this patient the MPS tracer uptake scores at stress are used.
n = number of segments in each MPS tracer uptake score group.

Copyright American Society of Nuclear Cardiology

Journal of Nuclear Cardiology | Official Journal of the American Society of Nuclear Cardiology

CONCLUSIONS

The typical uptake pattern seen on MPS in some patients with LBBB is likely related to underlying regional myocardial dyskinesia, wall thickening and wall thickness rather than stress-induced ischemia, myocardial fibrosis or specific ECG characteristics.

For Peer Review

Copyright American Society of Nuclear Cardiology
