# Supplementary figures and images for: To what extent are perfusion defects seen by myocardial perfusion SPECT in patients with left bundle branch block related to myocardial infarction, ECG characteristics, and myocardial wall motion?
Source: J Nucl Cardiol. 2020 May 25;28(6):2910–22. doi: 10.1007/s12350-020-02180-7 (PMC8709823; doi:10.1007/s12350-020-02180-7)

**Appendix**


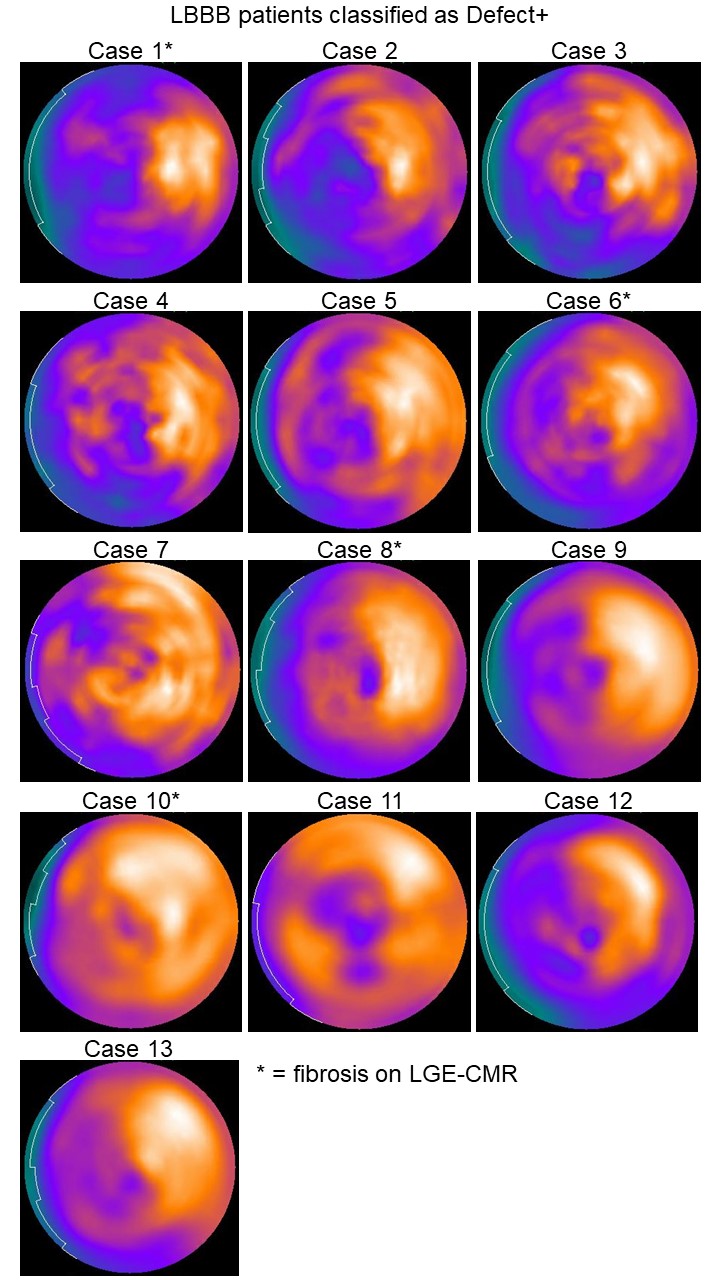


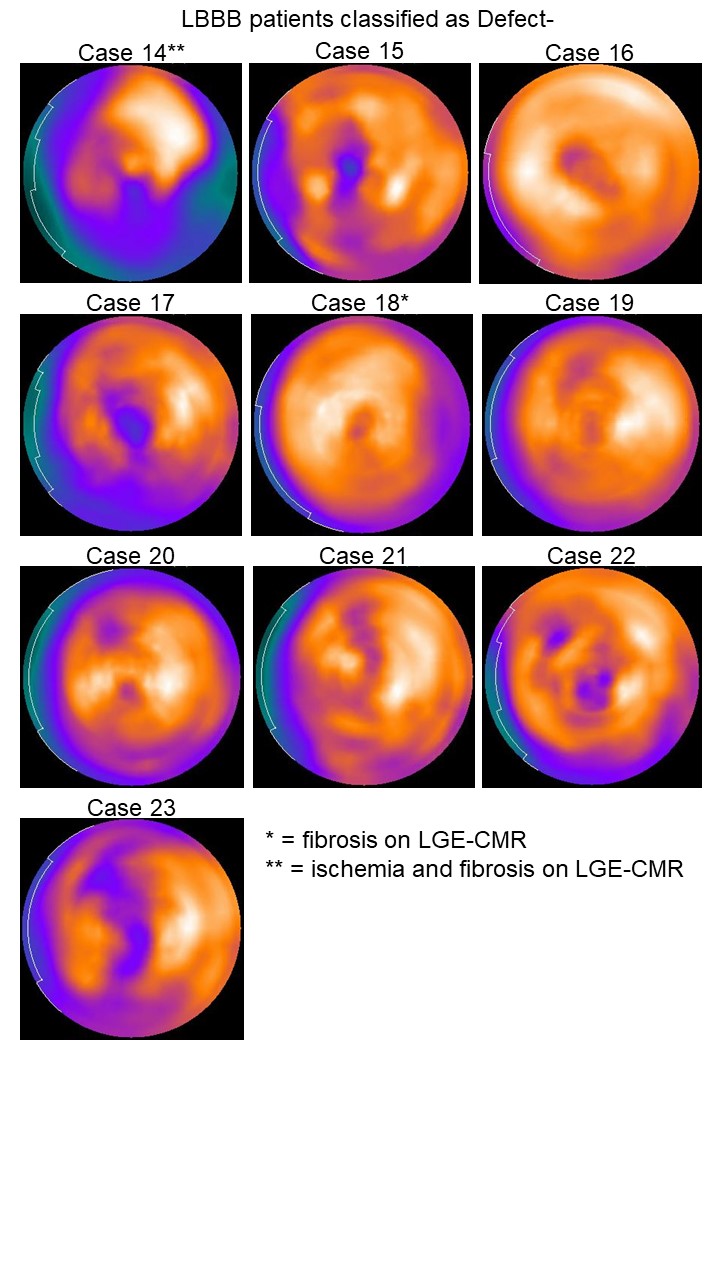

Supplement: Supplementary file 2 — Electronic supplementary material 2 (DOCX 317 kb) [file 12350_2020_2180_MOESM2_ESM.docx]
